# Altered caudate connectivity is associated with executive dysfunction after traumatic brain injury

**DOI:** 10.1093/brain/awx309

**Published:** 2017-11-23

**Authors:** Sara De Simoni, Peter O Jenkins, Niall J Bourke, Jessica J Fleminger, Peter J Hellyer, Amy E Jolly, Maneesh C Patel, James H Cole, Robert Leech, David J Sharp

**Affiliations:** 1 Computational, Cognitive and Clinical Neuroimaging Laboratory, Imperial College London, Division of Brain Sciences, Hammersmith Hospital, London, UK; 2 Department of Bioengineering, Imperial College London, London, UK; 3 Department of Imaging, Charing Cross Hospital, London, UK

**Keywords:** traumatic brain injury, functional connectivity, executive dysfunction, corticostriatal, anterior cingulate cortex

## Abstract

Traumatic brain injury often produces executive dysfunction. This characteristic cognitive impairment often causes long-term problems with behaviour and personality. Frontal lobe injuries are associated with executive dysfunction, but it is unclear how these injuries relate to corticostriatal interactions that are known to play an important role in behavioural control. We hypothesized that executive dysfunction after traumatic brain injury would be associated with abnormal corticostriatal interactions, a question that has not previously been investigated. We used structural and functional MRI measures of connectivity to investigate this. Corticostriatal functional connectivity in healthy individuals was initially defined using a data-driven approach. A constrained independent component analysis approach was applied in 100 healthy adult dataset from the Human Connectome Project. Diffusion tractography was also performed to generate white matter tracts. The output of this analysis was used to compare corticostriatal functional connectivity and structural integrity between groups of 42 patients with traumatic brain injury and 21 age-matched controls. Subdivisions of the caudate and putamen had distinct patterns of functional connectivity. Traumatic brain injury patients showed disruption to functional connectivity between the caudate and a distributed set of cortical regions, including the anterior cingulate cortex. Cognitive impairments in the patients were mainly seen in processing speed and executive function, as well as increased levels of apathy and fatigue. Abnormalities of caudate functional connectivity correlated with these cognitive impairments, with reductions in right caudate connectivity associated with increased executive dysfunction, information processing speed and memory impairment. Structural connectivity, measured using diffusion tensor imaging between the caudate and anterior cingulate cortex was impaired and this also correlated with measures of executive dysfunction. We show for the first time that altered subcortical connectivity is associated with large-scale network disruption in traumatic brain injury and that this disruption is related to the cognitive impairments seen in these patients.

## Teaser

Traumatic brain injury often produces executive dysfunction, causing long-term problems with behaviour and personality. De Simoni *et al.* report that executive dysfunction following TBI is associated with altered corticostriatal interactions important for behavioural control. These findings provide a target for the evaluation of treatments aimed at improving executive function after TBI.

## Introduction

Traumatic brain injury (TBI) commonly causes persistent cognitive and behavioural impairments, including executive dysfunction (Draper and Ponsford, 2008). While these executive problems have been linked to focal frontal lobe injury (Damasio *et al.*, 1994; Stuss and Alexander, 2007), dysexecutive problems are often seen in the absence of focal injury after TBI (Anderson *et al.*, 1995; Bigler, 2001). This cognitive impairment is associated with reduced structural integrity of cortico-subcortical connections; however, it is unclear how it relates to the functional interactions of the frontal lobes with subcortical structures that are linked to behavioural control (Little *et al.*, 2010; Leunissen *et al.*, 2013, 2014). Here we used advanced MRI techniques to investigate corticostriatal interactions after TBI, to test whether functional and structural disruption of this circuitry is associated with cognitive impairments.

Cognitive control processes that underpin executive function depend on the efficient functioning of corticostriatal circuits, which are often abnormal in other conditions that are characterized by executive dysfunction (Mennes *et al.*, 2011; Unschuld *et al.*, 2012). These circuits integrate both cognitive and motivational cortical information to facilitate motor control involved in goal-directed behaviours (Haber, 2016). As TBI disrupts brain network function and striatal physiology (Donnemiller *et al.*, 2000; Wagner *et al.*, 2005; Bonnelle *et al.*, 2011; Sharp *et al.*, 2011), one possibility that has not previously been investigated is that executive dysfunction after TBI is specifically associated with abnormal corticostriatal functional interactions. The majority of cortical input to the basal ganglia passes through the striatum (Haber, 2003). The region is organized into subregions including the caudate, putamen and nucleus accumbens, primarily associated with cognitive, motor and motivational processes, respectively (Haber, 2003).

Disruption to corticostriatal loops produces executive dysfunction in other diseases. For example, Huntington’s disease is characterized by progressive degeneration of the striatum, originating in the caudate (Douaud *et al.*, 2006). Prodromal Huntington’s disease patients demonstrate poor motor and executive function, together with disrupted functional and structural connectivity between cortical areas and the caudate (Unschuld *et al.*, 2012; McColgan *et al.*, 2015). Caudate connectivity is also related to executive function in attention deficit hyperactivity disorder (ADHD), which has been demonstrated using response inhibition and cognitive switching tasks (Mennes *et al.*, 2011). These studies suggest executive dysfunction after TBI could also be the result of altered corticostriatal connectivity, particularly in light of the effects of diffuse axonal injury and the susceptibility of neuromodulatory control systems such as dopamine that influence striatal function after TBI (Jenkins *et al.*, 2016).

The functional organization of corticostriatal connections can be investigated using resting-state functional MRI (Postuma and Dagher, 2006; Di Martino *et al.*, 2008; Barnes *et al.*, 2010; Choi *et al.*, 2012). This can be used to derive the functional connectivity between regions, which provides an estimate of the strength of their interactions. Specific subdivisions of the striatum are known to be connected to cortical networks, which reflect their function (Pauli *et al.*, 2016). Originally these circuits were thought to be largely segregated, with information processed in a parallel fashion (Alexander *et al.*, 1986). More recently it has become clear that basal ganglia connectivity also incorporates a degree of integration (Haber and Knutson, 2010; Bell and Shine, 2016). This configuration allows circuits to interface and produce complex cognitive control behaviours that rely on combining both cognitive and motivational information (Haber and Knutson, 2010). We have previously used a multivariate data-driven approach to identifying complex patterns of functional connectivity (Leech *et al.*, 2011, 2012). This method is well suited to identifying overlapping but distinct patterns of connectivity in regions that show complex functional connectivity, such as the brain’s hub regions that integrate processing from diverse cortical regions (Leech *et al.*, 2012). The method avoids the use of *a priori*-defined striatal or cortical seed regions to perform connectivity analyses and instead isolates independent signals and their corresponding spatial locations in a data-driven manner by performing an independent component analysis on the signal within the region of interest.

We have previously used resting-state functional connectivity to investigate the relationship between cognitive impairment and network dysfunction after TBI. Sustained attention impairments are associated with disruption to functional interactions within the default mode network (DMN; Bonnelle *et al.*, 2011). Abnormal interactions between the DMN and medial temporal lobes are also related to the extent of associative memory impairments following TBI (De Simoni *et al.*, 2016). These changes are often associated with underlying disruption of the structural connections produced by diffuse axonal injury, which can be measured using diffusion tensor imaging (DTI). For example, DTI measures of the salience network have been related to efficient DMN functioning (Bonnelle *et al.*, 2012). The combination of this multi-modal imaging provides a rich description of network dysfunction, which we apply here to study altered interactions between the cortex and subcortical structures following TBI for the first time.

Here we use a masked independent component analysis (mICA) approach to examine striatal functional connectivity after TBI. First we defined patterns of corticostriatal functional connectivity in a large healthy dataset (Human Connectome Project, HCP). These data have high spatial and temporal resolution, which for the first time allows striatal connectivity patterns to be separated in a data-driven way. This analysis provided detailed maps of corticostriatal interactions, which we used to compare functional connectivity between TBI patients and age-matched controls. In addition, we used DTI data to test whether altered functional connectivity between frontal and striatal structures was associated with damage to the tracts connecting them, which would potentially imply a causative role for diffuse axonal injury in the observed network dysfunction. We tested the following specific hypotheses: (i) corticostriatal functional connectivity is abnormally low following TBI; (ii) disruption in corticostriatal connectivity correlates with impairments in cognitive function; and (iii) abnormalities in striatal functional connectivity correlates with damage to corticalstriatal tracts.

## Materials and methods

### Participant demographics and clinical details

Forty-two patients (five females, mean age 40.6 ± 11.7 years, range 20–65) following a moderate–severe TBI at least 6 months previously were recruited from neurology clinics. Mean time since injury was 73.1 months ( ± 86.9, range 6–366 months). TBI severity was assessed according to the Mayo Classification (Malec *et al.*, 2007). All patients were identified as having persistent cognitive problems by the patient themselves, treating clinician or caregiver. Inclusion criteria were: age 20–65 years, no significant premorbid psychiatric or neurological history, alcohol or substance misuse, or significant previous TBI. Exclusion criteria included contraindication to MRI and a positive urine drug screen. Written, informed consent was obtained from all patients in accordance with the Declaration of Helsinki. The study was approved by the West London and GTAC Research Ethics Committee (14/LO/0067). Six patients were excluded from the functional imaging analyses based on stringent criteria for assessing the possible impact of movement during the resting-state scan (Supplementary material and ‘Control analyses’ section). Thirty-six patients were thus included in the functional imaging analysis. Diffusion and neuropsychological analysis was performed with the full dataset of 42 patients.

Twenty-one age and gender-matched healthy controls were recruited (four females, mean age 39.2 ± 12.1, range 21–61). Participants had no history of psychiatric or neurological illness, previous TBI, alcohol or substance misuse. All participants gave written informed consent. One healthy control was excluded from the functional imaging analyses due to movement during the resting-state scan (Supplementary material and ‘Control analyses’ section). Twenty controls were thus included in the functional imaging analysis. Diffusion and neuropsychological analysis was performed with the full dataset of 21 controls.

### Neuropsychological and well-being assessment

A standardized neuropsychological battery was used to assess cognitive function, including tests previously shown to be sensitive to TBI-related deficits (Table 1) (Kinnunen *et al.*, 2011). The Trail Making Test (TMT) and Delis-Kaplan Executive Function System (D-KEFS) Colour-Word Interference Test (Stroop) assessed information processing speed and executive function, including inhibition and task switching (Delis *et al.*, 2001); Wechsler Memory Scale (WMS-III) logical memory subtests I and II and the People Test (PT) from the Doors and People Test measured episodic memory (Wechsler, 1945); the Wechsler Abbreviated Scale for Intelligence (WASI) Matrix Reasoning and Wechsler Test of Adult Reading (WTAR) assessed reasoning ability and premorbid IQ, respectively (Wechsler, 1945). A laptop-based choice reaction time (CRT) task measured basic information processing speed.

**Table 1:** Neuropsychological measures in healthy controls and traumatic brain injury patients

**Cognitive domain**	**Neuropsychological test**	**HC Mean ( ± SD) *n = *21**	**TBI patients Mean (±SD) *n = *42**	**W**	***P***
**Processing speed**	Trail Making Test A (s)	19.4 (6.0)	32.1 (19.3)	242	0.002^a^
	Trail Making Test B (s)	45.8 (26.0)	69.1 (35.7)	223	<0.001^a^
	Stroop Colour Naming and Word Reading Composite Score (s)	24.1 (4.44)	30.9 (7.21)	182.5	<0.001^a^
	CRT Median RT (s)	0.38 (0.08)	0.43 (0.06)	270	0.008
	CRT RT SD (s)	0.07 (0.03)	0.08 (0.03)	321.5	0.053
**Executive function**	Trail Making Test B-A (s)	26.4 (22.3)	37.0 (23.0)	295	0.016
	Stroop Inhibition (s)	50.4 (12.1)	60.5 (14.2)	245.5	0.002^a^
	Stroop Inhibition-Switching (s)	56.7 (15.5)	71.0 (17.3)	218	<0.001^a^
	Stroop Inhibition-Switching versus Baseline Contrast (s)	33.05 (14.5)	40.5 (12.9)	272.5	0.007
**Memory**	WMS-III LM Immediate Recall (Total I)	49.5 (7.41)	35.1 (11.4)	746	<0.001^a^
	WMS-III LM Delayed Recall (Total II)	32.6 (6.83)	20.7 (10.7)	727.5	<0.001^a^
	WMS-III LM Retention	89.8 (9.6)	77.6 (25.2)	569.5	0.031
	WMS-III LM Recognition	26.6 (4.8)	25.1 (3.3)	621.5	0.004
	WMS-III LM Learning	5.6 (2.8)	3.6 (2.5)	633	0.002^a^
	PT Immediate Recall	30.0 (4.5)	22.6 (7.7)	718	<0.001^a^
	PT Delayed Recall	10.3 (2.9)	8.1 (3.4)	619	0.003
	PT Forgetting	1.5 (2.9)	2 (2.2)	326	0.035
**Intellectual ability**	WTAR Scaled	116.1 (6.7)	106.2 (11.5)	686.5	<0.001^a^
	WASI Matrix Reasoning	28.1 (4.7)	27.6 (4.5)	470.5	0.335

Independent sample *t-*tests were conducted using the Wilcoxon Rank Sum Test. ^a^Tests surviving Bonferroni correction for multiple comparisons.

CRT = choice reaction time; HC = healthy controls; PT = People’s Test; RT = reaction time; SD = standard deviation; WASI = Wechsler Abbreviated Scale for Intelligence; WMS-III = Wechsler Memory Scale, Third Edition; WTAR = Wechsler Test of Adult Reading.

Self-report measures were used to assess subjective well-being in all participants. The Lille Apathy Rating Scale (LARS) was used to assess apathy (Table 2) (Sockeel *et al.*, 2006); the Hospital Anxiety and Depression Scale (HADS) measured feelings of anxiety and depression. The visual analogue scale (VAS-F) evaluated fatigue severity on the day of scanning (Lee *et al.*, 1991). The Frontal Systems Behaviour Scale (FrSBe) was used in patients to evaluate subjective changes in behaviour post-injury (Table 2) (Grace and Malloy, 2001). Quality of life was assessed using the Short Form-36 (SF-36) (Table 2) (Ware and Sherbourne, 1992). Data were found to be non-normally distributed based on Shapiro-Wilk tests. Group differences in performance were investigated with the use of Wilcoxon Rank Sum Tests for independent samples (one-tailed) and Bonferroni corrected for multiple comparisons. Wilcoxon Signed-Rank tests for paired samples (one-tailed) were used to assess longitudinal changes in FrSBe scores.

**Table 2:** Assessments of subjective well-being in both healthy controls and traumatic brain injury patients

**Measure**	**Domain/subscale**	**Healthy controls Mean (±SD)**	**TBI patients Mean (±SD)**	**W**	***P***
**LARS**	Action initiation	−3.88 (0.3)	−2.91 (1.4)	234	<0.001^a^
	Intellectual curiosity	−3.73 (0.4)	−2.43 (1.6)	191	<0.001^a^
	Emotional responsiveness	−3.24 (0.7)	−2.24 (1.5)	278	0.009
	Self-awareness	−3.76 (0.6)	−2.88 (1.6)	294	0.007
	Total	−32.9 (2.8)	−23.1 (10.3)	124.5	<0.001^a^
**HADS**	Anxiety	5.55 (3.9)	7.41 (4.7)	312.5	0.067
	Depression	3.0 (2.7)	6.85 (5.3)	226	0.002^a^
**VAS-F**	Fatigue	26.9 (19.5)	44.2 (22.9)	235	0.002^a^
	Energy	58.4 (13.7)	43.8 (20.9)	608.5	0.002^a^
**SF-36**	Physical functioning	97.6 (4.1)	74.6 (24.1)	724.5	<0.001^a^
	Physical health	94.1 (17.5)	47.9 (40.9)	708	<0.001^a^
	Emotional problems	92.1 (20.8)	59.4 (43.1)	622.5	<0.001^a^
	Energy/fatigue	66.0 (17.4)	45.4 (23.5)	640.5	<0.001^a^
	Emotional well-being	75.6 (14.8)	65.6 (19.6)	566.5	0.021
	Social functioning	91.1 (14.9)	62.2 (24.8)	728.5	<0.001^a^
	Pain	92.4 (11.0)	79.2 (23.8)	564.5	0.017
	General health	79.3 (15.0)	62.6 (22.7)	624.5	0.002^a^
**FrSBe (Self)**	Apathy (pre)	NA	24.6 (6.0)	NA	NA
	Disinhibition (pre)	NA	27.0 (7.1)	NA	NA
	Executive function (pre)	NA	34.8 (7.3)	NA	NA
	Total (pre)	NA	85.9 (17.5)	NA	NA
	Apathy (post)	NA	35.8 (10.7)	21	<0.001^a^^,^^b^
	Disinhibition (post)	NA	33.2 (9.0)	92.5	<0.001^a^^,^^b^
	Executive function (post)	NA	44.5 (11.8)	59.5	<0.001^a^^,^^b^
	Total (post)	NA	113.0 (28.6)	40	<0.001^a^^,^^b^
**FrSBe (Other)**	Apathy (pre)	NA	23.9 (4.9)	NA	NA
	Disinhibition (pre)	NA	26.4 (7.5)	NA	NA
	Executive function (pre)	NA	34.5 (9.2)	NA	NA
	Total (pre)	NA	84.8 (18.4)	NA	NA
	Apathy (post)	NA	30.5 (8.4)	3	<0.001^a^^,^^b^
	Disinhibtion (post)	NA	30.3 (8.9)	32.5	<0.001^a^^,^^b^
	Executive function (post)	NA	43.3 (9.0)	11.5	<0.002^a^^,^^b^
	Total (post)	NA	104.2 (22.7)	15.5	<0.001^a^^,^^b^

Independent Sample *t-*tests were conducted using the Wilcoxon Rank Sum Test.

^a^Denotes tests surviving Bonferroni correction for multiple comparisons.

^b^Paired-sample Wilcoxon Signed-Rank test between pre-post injury FrSBe assessments.

FrSBe = Frontal Systems Behaviour Scale; HADS = Hospital Anxiety and Depression Scale; LARS = Lille Apathy Rating Scale (more negative scores indicates lower levels of apathy); NA = not applicable; SF-36 = Short Form-36; VAS-F = Visual Analogue Scale for Fatigue.

### Structural and functional MRI data acquisition

MRI data from the Human Connectome Project (http://www.humanconnectome.org/) were collected on a 3 T Siemens Connectome Skyra, including a T_1_-weighted MPRAGE structural scan, resting-state functional MRI and DTI using multiband acquisitions.

MRI data from a separate cohort of TBI patients and healthy control subjects, were acquired using a 3 T Siemens Magnetom Verio Syngo with a 32-channel head coil. Standard clinical MRI was collected. Resting-state functional MRI data were also acquired, alongside a high-resolution T_1_-weighted image and DTI (see Supplementary material for details on the acquisition parameters for both cohorts).

### MRI data analysis

Striatal subdivisions, corticostriatal functional and structural connectivity was defined on a large independent dataset with high spatial and temporal resolution taken from the HCP. These results were then used to assess group differences in corticostriatal connectivity in a separate sample of healthy controls and TBI patients. Structural connectivity analysis was informed by the functional connectivity results in the TBI patients. Disruption of functional connectivity was particularly observed between caudate subdivisions and the cingulate cortex (see ‘Results’ section). Therefore, to investigate whether abnormalities in caudate functional connectivity were associated with damage to relevant white matter tracts, the HCP dataset was used to define tracts extending from the caudate to the anterior cingulate cortex (ACC).

#### Human Connectome Project analysis

##### Preprocessing

Data for 100 randomly selected healthy participants (41 males, age range: 22–36) from the HCP were analysed. Preprocessing of both resting-state functional MRI and diffusion data was performed by the HCP consortium with FMRIB Software Library (FSL) and Freesurfer (version 5.2). A subset of 94 healthy participants from this HCP cohort was used for the tractography analysis. For details on preprocessing procedures see Supplementary material (see also Glasser *et al.*, 2013; Smith *et al.*, 2013; Van Essen *et al.*, 2013).

##### Defining striatal subdivisions based on functional signals

**Figure 1:**
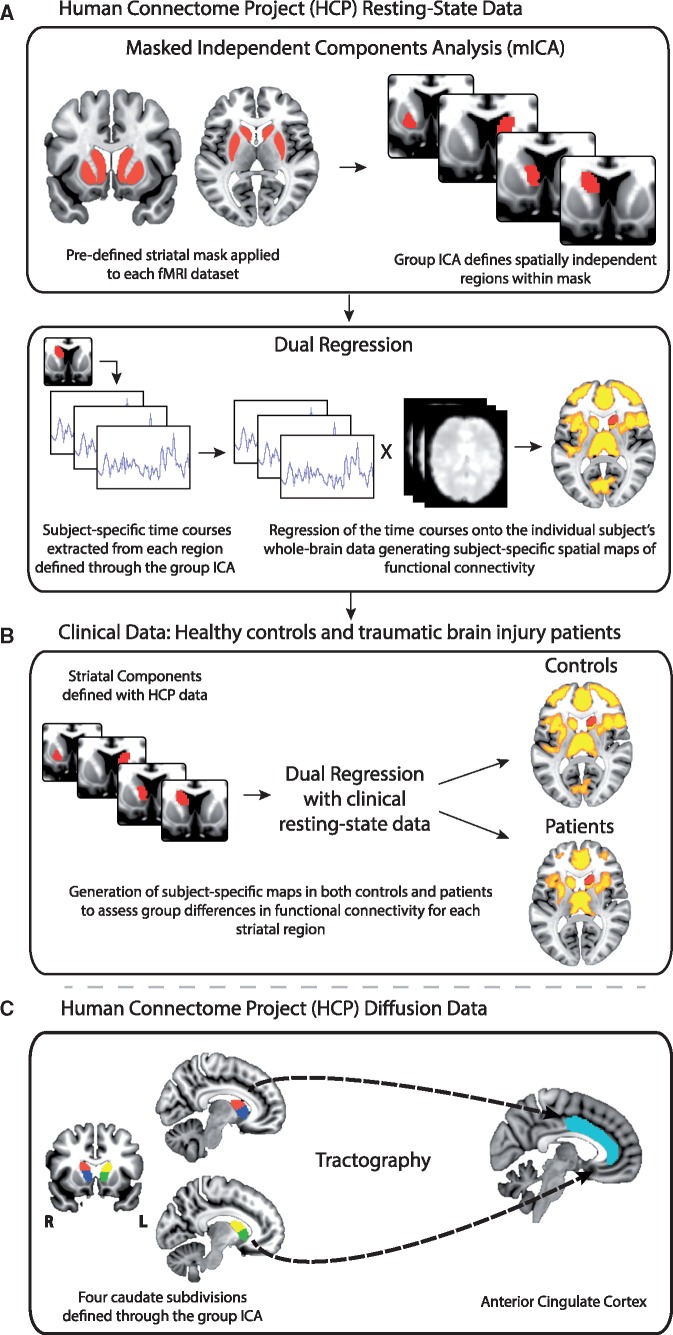
**Overview of the imaging methods used to assess corticostriatal connections.** (**A**) A combined mICA and dual-regression approach was used to define corticostriatal functional connectivity networks within the HCP resting-state data. MICA was performed and constrained to extract independent components within a striatal mask. Corticostriatal functional connectivity of the individual independent components was assessed with dual regression. (**B**) The independent components and associated corticostriatal functional connectivity networks defined with the HCP dataset were then used to evaluate functional connectivity differences in a clinical resting-state dataset including TBI patients and healthy controls. (**C**) Tractography analysis was performed with the HCP data between selected striatal independent components and the ACC. These white matter tracts were then used to assess differences in white matter integrity between TBI patients and controls.

**Figure 2:**
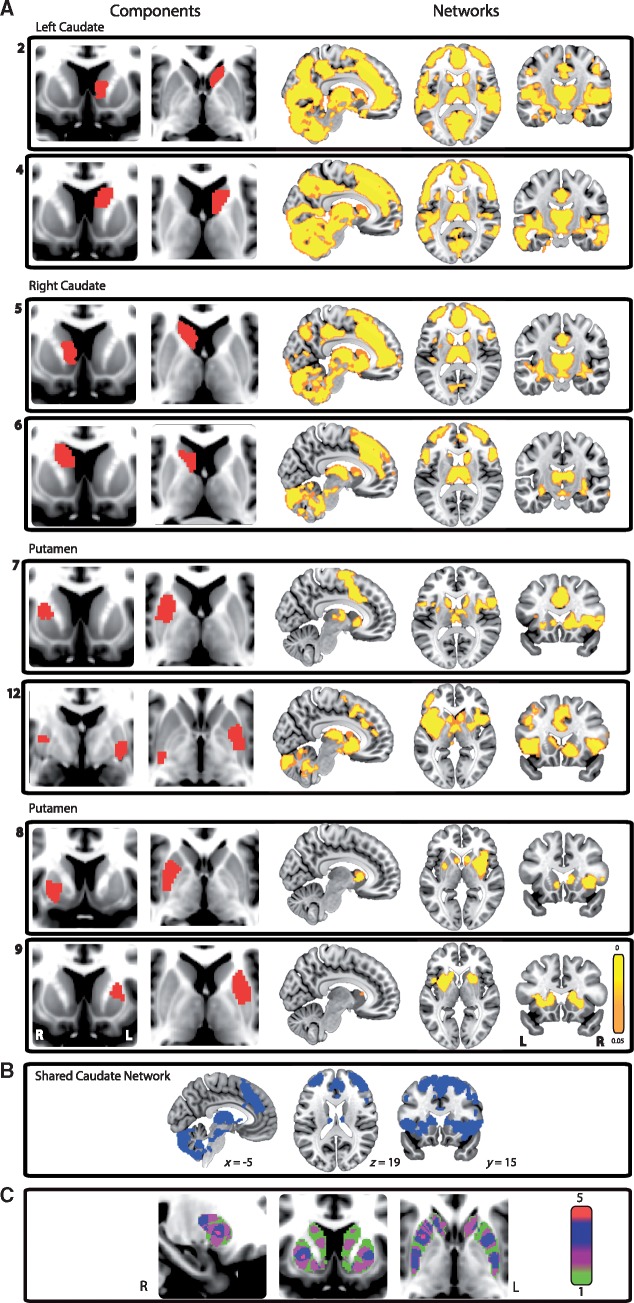
**Subdivisions of the striatum and their associated corticostriatal networks.** (**A**) Eight of the 12 striatal subdivisions (*left*) and their associated functional connectivity networks (*right*) derived from the HCP data. All networks are thresholded at *P < *0.05, FWE corrected for multiple comparisons. (**B**) A map displaying the brain areas common across the four caudate networks. (**C**) An overlap map of the striatal subdivisions. Red indicates areas of highest overlap, which are found in the right putamen and nucleus accumbens. Green indicates areas of lowest overlap.

MICA was performed with the mICA Toolbox on the HCP preprocessed data (Moher Alsady *et al.*, 2016; Fig. 1A). MICA was constrained to extract independent components within a striatal mask, defined *a priori* using the Harvard-Oxford probabilistic anatomical atlas within FSL (thresholded at >50%). This pipeline allows the HCP data to be spatially smoothed using a 6 mm kernel after masking, avoiding signal contamination from outside the striatum. MICA was run to extract 12 independent components (DIM12), based on previous work, which identified 12 corticostriatal functional networks (Di Martino *et al.*, 2008). To establish whether decomposing striatal signal into 12 independent components is representative of the underlying data, mICA was also run using dimensionalities in the range of 2–20. Spatial cross-correlations were performed between dimensionalities to assess the stability of components. High spatial correlations would suggest that DIM12 components are identifiable and robust across various ICA decompositions. In addition, to determine the degree to which the DIM12 striatal components were spatially separable, pairwise correlations were performed between each pair of components. Low correlations suggest good spatial separation. Subsequently, the striatum was parcellated into 12 subdivisions based on z-transformations of the ICA results, with voxels allocated to a particular subdivision based on the component with the highest z-score at that location (Fig. 2).

##### Defining corticostriatal functional networks with the Human Connectome Project data

A combined mICA and dual-regression approach was used to define corticostriatal functional connectivity networks within the HCP resting-state functional MRI data (Bonnelle *et al.*, 2011; Sharp *et al.*, 2011; Leech *et al.*, 2012; Fig. 1A). This approach provides a voxel-wise measure of functional connectivity that represents the temporal correlation between each voxel and the activity of a region or network of interest (Sharp *et al.*, 2011). Details of the method can be found in our previous work (Sharp *et al.*, 2011; Leech *et al.*, 2012). To define corticostriatal functional networks within the HCP cohort, 12 group-level striatal independent components from the mICA procedure described above were used to produce subject-specific corticostriatal functional connectivity networks (Fig. 1A). A permutation-based one-sample *t*-test was then run to generate group-level maps of functional connectivity for each component. Functional connectivity networks were defined using non-parametric permutation testing, thresholded using the threshold-free cluster enhancement (TFCE) method and corrected for multiple comparisons with a family-wise error (FWE) rate of *P < *0.05. These analyses were constrained within a grey matter mask, provided through FSL. Resulting networks were thresholded and binarized to investigate between group differences in corticostriatal functional connectivity between TBI and control datasets (Fig. 1B).

##### Defining caudate to anterior cingulate cortex white matter tracts with the Human Connectome Project data

Tractography was performed using MRtrix (Tournier *et al.*, 2012). This framework combines estimation of fibre orientations using constrained spherical deconvolution (CSD) with a probabilistic streamlines approach to define structural connectivity. Four separate regions of interest were used to drive the tractography, consisting of the four caudate subdivisions of the striatal parcellation (Figs 1 and 5). Streamlines (*n* = 3000) seeded within these regions of interest were generated from each subject’s fibre orientation density image and terminated using an ACC mask, defined *a priori* using the Harvard-Oxford atlas (thresholded at >50%). Fibre tracking was also terminated if the curvature angle was >45°. Each individual’s tract density image was thresholded to remove voxels containing <5% of total number of streamlines. Tractograms were combined to create single tracts where >25% of subjects had data. This resulted in four white matter tracts: right anterior caudate to ACC, left anterior caudate to ACC, right posterior caudate to ACC and left posterior caudate to ACC.

To investigate the specificity of the findings to white matter tracts associated with caudate-ACC structural connections, additional ‘control’ tracts were also tested. These included (i) a striatum to primary motor cortex white matter tract defined within the HCP data; and (ii) tracts taken from the John Hopkins University White-Matter Tractography Atlas within FSL, including the corpus callosum (body, genu and splenium), anterior thalamic radiation, inferior fronto-occipital fasciculus and cingulo-hippocampal tract.

#### Clinical study data

##### Preprocessing

Data were analysed using FSL (Version 5.0.6; Smith *et al.*, 2004) (see Supplementary material for details on preprocessing of the resting-state functional MRI and diffusion data).

##### Defining differences in corticostriatal functional connectivity between controls and patients with TBI

A combined mICA and dual-regression approach was used to assess corticostriatal functional connectivity differences between patient and control groups (Bonnelle*et al.*, 2011; Sharp *et al.*, 2011; Fig. 1B). Twelve dual regressions were performed. For each dual regression a different striatal independent component was used to perform the analysis; each was constrained to voxels within the corresponding whole-brain network identified within the HCP cohort. Between-group effects were assessed using non-parametric permutation testing (Winkler *et al.*, 2014), thresholded using TFCE and FWE-corrected for multiple comparisons at *P < *0.05.

Measures of altered corticostriatal functional connectivity in TBI patients were correlated with neuropsychological and self-report measures to explore whether these changes were related to particular cognitive impairments identified in the patients. Spearman’s correlations (referred to in the results as rho) were used and corrected for multiple comparisons using a false-discovery rate (FDR) of *P < *0.05. Spearman partial correlations were also run to account for the effects of age and time since injury.

To investigate the specificity of post-traumatic changes in corticostriatal connectivity, we performed additional ‘control’ functional connectivity analyses to investigate whether thalamo-cortical and fronto-parietal connectivity was affected by TBI. Six dual regression analyses were performed. Four separate subthalamic regions of interest were used to examine thalamo-cortical connectivity. These were derived from the FSL Oxford Thalamic Connectivity Probability Atlas, with subdivisions of the thalamus defined on the basis of their white matter connectivity to cortical areas. We focused on bilateral thalamic subdivisions projecting to prefrontal and motor cortical areas, thresholded at 25% (Supplementary Fig. 2). In addition, fronto-parietal functional connectivity was investigated within left and right fronto-parietal intrinsic connectivity networks, defined from functional connectivity analysis of resting-state functional MRI data (Smith *et al.*, 2009). Functional connectivity was compared between TBI and control groups.

In addition, to investigate the specificity of subcortical-cortical interactions for executive function we also investigated whether thalamo-cortical functional connectivity to the ACC was related to behaviour. This particular analysis was motivated by the results of the cortico-striatal analysis, which was the main focus of the paper. Thalamic functional connectivity to the ACC was investigated using functional connectivity derived from the four thalamic subdivisions. For each of these analyses, mean functional connectivity values for each subject were extracted from the ACC (as defined above). Spearman’s correlation was used to test the relationship between mean functional connectivity values and all neuropsychological tests. This included measures shown to correlate with caudate functional connectivity as well as our other cognitive measures.

##### Defining differences in white matter tract structural connectivity between healthy controls and patients with TBI

A region of interest approach was used to assess white matter structure differences between TBI patients and healthy controls. Fractional anisotropy and mean diffusion metrics were extracted from the four caudate–ACC white matter tracts defined using the HCP data. Data were also extracted from the control tracts. Between-group differences were evaluated using linear mixed-effects models. Group was defined as the between-subjects factor, region of interest as the within-subjects factor (fixed effects) and subject was defined as a random effect to model variability in subject intercepts. Age was included as a covariate. *Post hoc t-*tests were used to investigate any significant main effects.

Diffusion metrics from each region of interest were correlated with measures of functional connectivity for the corresponding caudate subdivision to assess whether functional changes reflected the underlying structural variability. Correlations were also performed with information processing speed and executive function measures shown to be associated with changes in caudate functional connectivity. Spearman partial correlations were run to account for the effects of age and time since injury, and corrected for multiple comparisons using FDR of *P < *0.05. To assess whether combining functional connectivity and diffusion measures explained more of the variability in cognitive performance than either imaging measure alone, linear models were run. Variability was quantified by the residual sum of squares and models compared using F-tests. Specifically, functional connectivity and fractional anisotropy from the four caudate subdivisions and tracts, respectively were included in four separate models to assess variability in executive function (i.e. Stroop task performance).

##### Lesion analysis

Focal lesion masks were generated to determine lesion volume and create overlap maps. This was done to ensure that the functional connectivity results were not primarily driven by lesioned cortex. Lesions were also reported by a neuroradiologist (Supplementary material, Supplementary Fig. 1C and Supplementary Table 1).

## Results

### Neuropsychology

#### Cognitive impairment, executive dysfunction, apathy and fatigue after TBI

The TBI patients demonstrated cognitive impairments in measures of information processing speed, executive function and memory when compared with an age-matched control group (Table 1). Specifically, information processing speed and executive function as measured by both the Trail Making and Stroop, and memory impairments on the Logical Memory and Doors and People tests were impaired after correction for multiple comparisons (Table 1). The WTAR estimate of IQ was lower in the TBI group. Performance on other measures differed between groups but did not survive correction for multiple comparisons (Table 1).

TBI patients also reported increased apathy, which affected both the physical (action initiation) and cognitive (intellectual curiosity) domains, as measured by the LARS (Table 2). Increases in apathy, together with behavioural evidence of executive dysfunction were also apparent using the FrSBe (Table 2). These did not differ significantly from caregiver assessments, suggesting a good level of self-awareness in the patients (*P* > 0.5). Patients reported significant levels of fatigue and felt more depressed than healthy controls. Perceived general health was significantly lower than that reported by healthy controls (Table 2).

### Imaging

#### Distinct subdivisions of the striatum defined on the basis of their local functional connectivity

Striatal subdivisions were identified based on distinct patterns of local functional connectivity calculated from the HCP data. Twelve independent but spatially overlapping subdivisions were identified across the caudate nucleus, putamen and nucleus accumbens (Fig. 2A). Four subdivisions were located in anterior and posterior parts of the right and left caudate. Predominantly left and right lateralized putamen components were also identified. No distinct subdivisions within the nucleus accumbens were identified; however, both anterior caudate and two anterior putamen subdivisions extended into this region.

The spatial overlap between components was maximal within the right putamen and nucleus accumbens, where a maximum of five subdivisions overlapped (Fig. 2C). Spatial pairwise correlations were performed for each pair of components to establish the degree of spatial separation between subdivisions (Supplementary Fig. 1A). The highest spatial correlation coefficient was 0.11, which suggests that, although there is some spatial overlap, ICA successfully produced striatal subdivisions with reasonable spatial separation.

#### Striatal subdivisions are associated with distinct corticostriatal networks

Next we tested how activity in the striatal subdivisions correlated with cortical activity (Fig. 2A). The four caudate subdivisions showed similar patterns of functional connectivity, which included the anterior cingulate and paracingulate gyri, as well as the thalamus, insula, cerebellum, supramarginal gyrus, superior and inferior frontal gyri (Fig. 2B). Putamen subdivisions were associated with more restricted networks including the caudate, insular cortex, thalamus, cingulate and paracingulate gyri and motor cortical areas.

#### Caudate functional connectivity is reduced following traumatic brain injury

**Figure 3:**
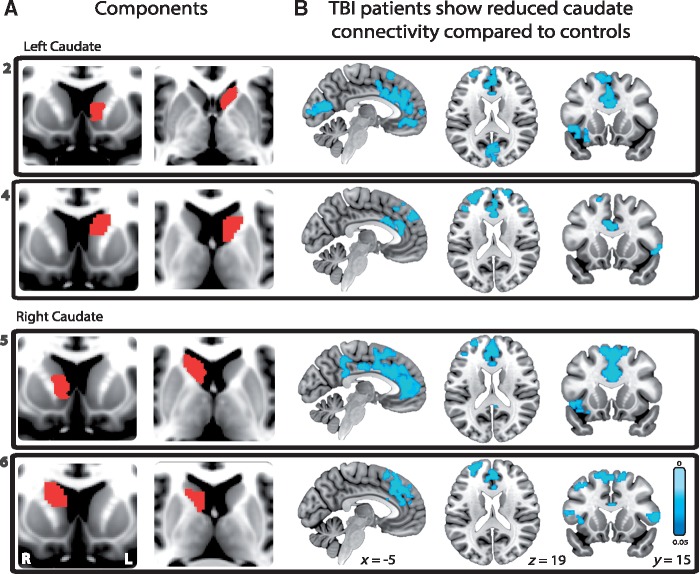
**Corticostriatal functional connectivity differences between TBI patients and healthy controls.** (**A**) Four striatal subdivisions located in bilateral anterior (subdivisions 2 and 5) and posterior caudate (subdivisions 4 and 6) together with (**B**) the associated reductions in functional connectivity in TBI patients compared to healthy controls. For each caudate subdivision the functional connectivity analysis was constrained to voxels within the corresponding whole-brain network identified within the HCP cohort. All results are thresholded at *P < *0.05, FWE corrected for multiple comparisons.

Next we investigated whether striatal functional connectivity was affected by TBI. Caudate functional connectivity was reduced in the TBI group (Fig. 3). All four subdivisions of the caudate showed reduced cortical functional connectivity in patients compared to controls. A similar pattern of abnormal functional connectivity was seen across these subdivisions. Reduced striatal functional connectivity to large parts of the anterior cingulate cortex, extending into the superior frontal gyrus was observed. In addition, the right anterior caudate subdivision showed reduced functional connectivity to the posterior cingulate cortex extending into the precuneus. Reduced functional connectivity to parts of the inferior and middle frontal gyri and insulae bilaterally was also seen (Fig. 3). No other striatal subdivisions showed significant changes in functional connectivity between patients and controls. In addition, thalamo-cortical and fronto-parietal connectivity was not significantly different in patients compared to controls (Supplementary Fig. 2B).

#### Right caudate functional connectivity is associated with executive dysfunction and fatigue

**Figure 4:**
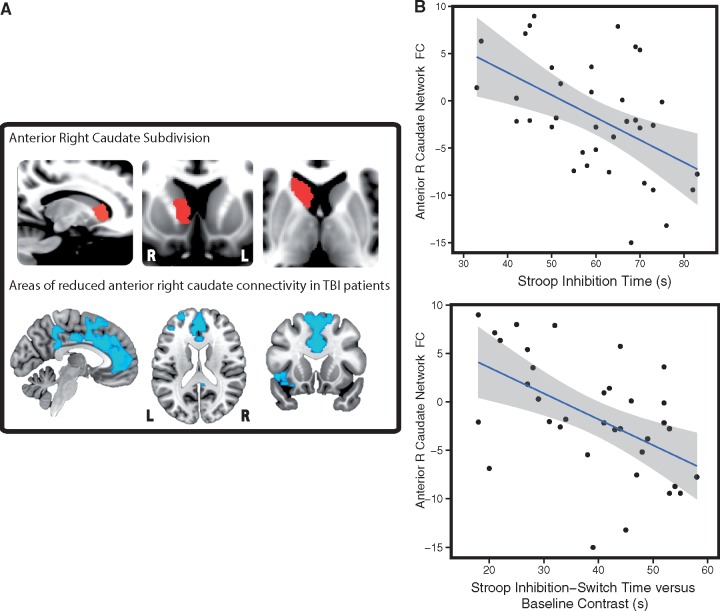
**The relationship between corticostriatal functional connectivity and neuropsychological measures.** (**A**) The anterior right caudate subdivision (*top*) and associated reductions in functional connectivity (FC) in TBI patients. The functional connectivity map is thresholded at *P < *0.05, FWE corrected for multiple comparisons. (**B**) Graphs representing significant correlations (*P < *0.01, FDR corrected) between right anterior caudate functional connectivity and performance on the Stroop task, including measures of executive function corrected for baseline speed. Reduced functional connectivity was correlated with slower reactions times. Areas highlighted in grey correspond to the 95% confidence intervals. L = left; R = right.

Abnormal caudate functional connectivity was correlated with cognitive impairment (Fig. 4B). After correcting for multiple comparisons, the right anterior caudate functional connectivity correlated with information processing speed measures both on the Trail Making (rho = −0.408, *P = *0.041) and Stroop task (Composite baseline score: rho = −0.411, *P = *0.041; Inhibition: rho = −0.517, *P = *0.008; Switching: rho = −0.605, *P = *0.001) (Fig. 4B). In addition, connectivity in this region was correlated with a measure of executive function corrected for baseline speed (Inhibition-Switching versus Baseline Contrast: rho = −0.592, *P = *0.001; Fig. 4B) and with a measure of memory function (delayed recall on the People’s Test, rho = 0.421, *P = *0.047). Right posterior caudate functional connectivity correlated with information processing speed and executive function measures on the Stroop task (Inhibition: rho = −0.492, *P = *0.02; Switching: rho = −0.508, *P = *0.02; Inhibition-Switching versus Baseline Contrast: rho = −0.472, *P = *0.021). Left caudate connectivity was not correlated with neuropsychological measures. Similar results were found with and without age and time since injury correction. No significant relationships were found in healthy control subjects.

Right anterior caudate functional connectivity was also significantly correlated with increased levels of fatigue on the day of testing, as measured by the VAS-F (rho = −0.444, *P = *0.013). Increased connectivity of the left anterior caudate was associated with an increase in subjective feelings of apathy and loss of energy post-injury, as assessed by the FrSBe (rho = 0.396, *P = *0.017 uncorrected) and VAS-F (rho = −0.366, *P = *0.03 uncorrected) respectively. These correlations were non-significant when correcting for multiple comparisons. No other significant correlations were found between caudate connectivity and self-report measures.

Functional connectivity between the prefrontal or primary motor thalamic subdivisions to the ACC was not correlated with any neuropsychological measures, following multiple comparison correction. In addition, measures shown to correlate with caudate functional connectivity, such as executive function, processing speed and delayed recall, were not correlated with thalamic-ACC functional connectivity even if multiple comparison correction was not performed (Supplementary Table 2 andSupplementary Fig. 2).

#### Caudate to ACC structural connectivity is abnormal following TBI

**Figure 5:**
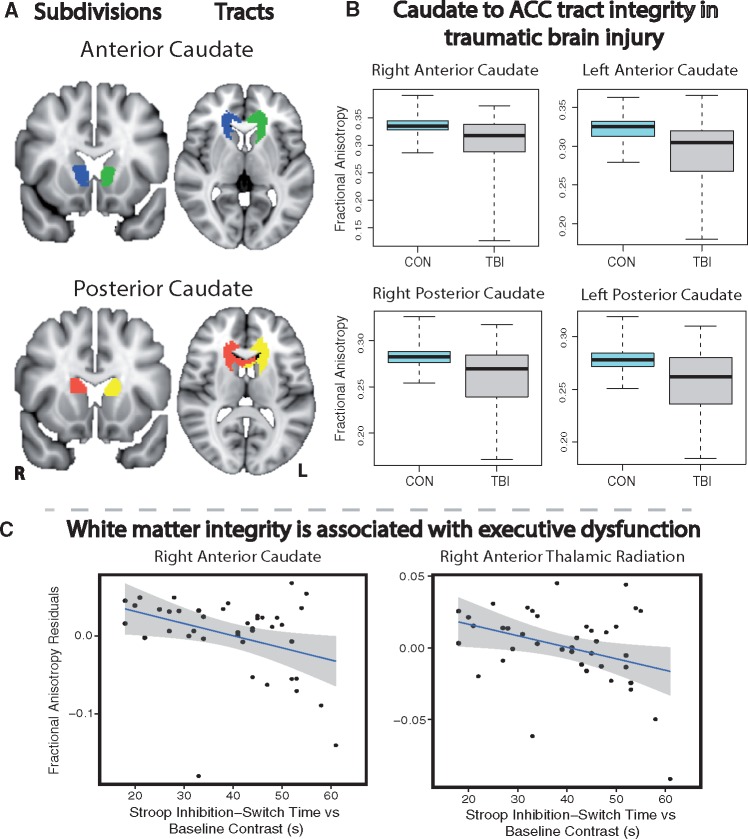
**Structural connections between the caudate subdivisions and the ACC.** (**A**) Anterior and posterior caudate subdivisions together with the white matter tracts connecting them to the ACC, defined using the HCP data. (**B**) Differences in caudate-ACC white matter integrity between patients with TBI and healthy controls (CON). (**C**) Graphs representing correlations between fractional anisotropy measures in two white matter tracts and performance on the Stroop task executive function measure corrected for baseline speed. Reductions in white matter integrity were correlated with slower reaction times. Spearman’s partial correlations were significant at *P < *0.05, FDR corrected for multiple comparisons. Fractional anisotropy measures have been plotted with variance for age partialled out.

Next we investigated whether abnormalities in functional connectivity were associated with underlying white matter disruption (Fig. 5). We focused this analysis on the structural connections between the caudate subdivisions and the ACC, because these regions showed a common pattern of abnormal functional connectivity that correlated with cognitive performance (Fig. 5A). TBI patients showed microstructural abnormalities in the four tracts connecting the ACC to caudate subdivisions (Fig. 5B). A significant group effect was present for both fractional anisotropy [*F*(1,60) = 10.61, *P = *0.002] and mean diffusivity [*F*(1,60) = 7.63, *P = *0.008]. *Post hoc t*-tests demonstrated that fractional anisotropy was reduced and mean diffusivity was increased in all tracts (*P < *0.05, FDR corrected). After correction for multiple comparisons and partialling out the effects of age and time since injury, fractional anisotropy and mean diffusivity measures correlated with information processing speed and executive function measures (Table 3 and Fig. 5). Specifically, fractional anisotropy within the right caudate to ACC tracts (anterior and posterior) were correlated with both measures of information processing and executive function corrected for baseline speed (*P < *0.05; Fig. 5C), whereas only the left posterior caudate tract was correlated with these measures (*P < *0.05). Mean diffusivity measures were correlated with information processing and executive function within the posterior right and left caudate tracts (*P < *0.05) (Table 3). No significant relationships were found in healthy controls.

**Table 3:** Relationship between neuropsychological measures and diffusion metrics in TBI patients

Cognitive domain	Neuropsychological test	Caudate subdivision							
		Fractional anisotropy, rho (*P*-value)				Mean diffusivity, rho (*P*-value)			
		Right anterior	Right posterior	Left anterior	Left posterior	Right anterior	Right posterior	Left anterior	Left posterior
**Processing speed**	Stroop Colour Naming and Word Reading Composite Score (s)	−0.212 (0.180)	−0.332 (0.030)	−0.168 (0.293)	−0.258 (0.100)	0.161 (0.315)	0.260 (0.097)	0.110 (0.497)	0.261 (0.095)
**Executive function**	Stroop Inhibition (s)	−0.323 (0.047)	−0.404 (0.009)	−0.245 (0.159)	−0.358 (0.024)	0.256 (0.137)	0.314 (0.055)	0.189 (0.314)	0.230 (0.070)
	Stroop Inhibition- Switching (s)	−0.410 (0.011)	−0.495 (0.002)	−0.319 (0.077)	−0.415 (0.020)	0.324 (0.070)	0.448 (0.004)	0.248 (0.228)	0.412 (0.014)
	Stroop Inhibition- Switching versus Baseline Contrast (s)	−0.431 (0.011)	−0.433 (0.006)	−0.322 (0.077)	−0.365 (0.024)	0.363 (0.065)	0.451 (0.004)	0.292 (0.228)	0.402 (0.014)

Correlations were performed using Spearman’s rank-order approach (rho) accounting for age and time since injury. *P*-values are adjusted for multiple comparisons using a false-discovery rate (FDR) of *P < *0.05.

Interestingly, there was no correlation between measures of caudate structural integrity and functional connectivity, despite the fact that they both correlated with similar cognitive measures. To assess the relative contributions of fractional anisotropy and functional connectivity to executive function, linear models including both measures were run. For three of the four caudate subdivisions/tracts, including both these measures explained more variability than just including fractional anisotropy (*P < *0.05). However, for the right caudate subdivisions/tracts, the combined models did not explain significantly more variance compared to including functional connectivity alone, suggesting that functional connectivity explains more of the variability in performance than fractional anisotropy.

Patients with TBI also showed microstructural abnormalities in control tracts, including the striatum to motor cortex, corpus callosum, anterior thalamic radiation, inferior fronto-occipital fasciculus and cingulo-hippocampal tracts. A significant group effect was present for both fractional anisotropy [*F*(1,60) = 14.47, *P = *0.0003] and mean diffusivity [*F*(1,60) = 12.36, *P = *0.0008]. Differences were present in all tracts except within the left cingulo-hippocampal tract. Information processing speed and executive function impairments were also correlated with changes in fractional anisotropy and mean diffusivity in control tracts. Following multiple comparison correction and accounting for the effects of age and time since injury, correlations with fractional anisotropy were restricted to the genu and splenium of the corpus callosum, anterior thalamic radiation and inferior fronto-occipital fasciculus (*P < *0.05; Fig. 5C). In contrast, correlations with mean diffusivity changes were seen in the striatum to motor cortex tract, genu and splenium of the corpus callosum, thalamic radiation, right inferior fronto-occipital fasciculus and cingulo-hippocampal tract (*P < *0.05).

### Control analyses

#### Motion

We were careful to ensure that motion artefacts did not contaminate our estimate of functional connectivity. Six patients and one healthy control exceeded criteria for excessive motion and were thus excluded from further imaging analysis (Supplementary material). All remaining participants’ data were de-noised using FSL FIX. This controlled for any residual effects of motion on functional connectivity. Motion was minimal across all the remaining participants (<0.31 mm in mean frame-wise displacement). Following these exclusions, there was no evidence for group differences in motion and no evidence that motion correlated with functional connectivity. No significant differences in mean frame-wise displacement were found between groups [*t*(39.8) = 1.5, *P = *0.151], with TBI patients and healthy controls demonstrating an average of 0.16 mm and 0.14 mm, respectively. No significant differences in blood oxygen level-dependent signal intensity (DVARS) between groups were found [*t*(38.05) = −1.33, *P = *0.192]. There were also no correlations between caudate functional connectivity and mean frame-wise displacement (*P* > 0.3 for all four subdivisions).

#### Stability of the striatal subdivisions across different ICA dimensionalities

ICA can be performed at different dimensionalities i.e. different numbers of striatal subdivisions can be derived (Supplementary Fig. 1B). We tested whether the 12 subdivisions we studied were reproduced at different dimensionalities. An ICA was run at dimensionalities ranging from 2 to 20. Spatial correlations were then performed between each of the 12 striatal subdivisions derived from the original ICA (DIM12) and all components resulting from the additional ICA dimensionalities. The spatial correlations suggest that the striatal subdivisions derived from DIM12 can be stably identified when dimensionalities above ∼10 are used (Supplementary Fig. 1B). This stability provides reassurance that decomposing striatal signal into 12 independent components is representative of the underlying data (Smith *et al.*, 2009; Leech *et al.*, 2011; Geranmayeh *et al.*, 2014).

## Discussion

For the first time we show that patients with cognitive impairment after TBI show abnormal patterns of striatal functional connectivity. Within the striatum, these were only seen in the connectivity of subdivisions of the caudate, where reduced functional connectivity to large-scale cortical and subcortical networks was observed. Distinct patterns of functional connectivity were seen in other striatal regions, but these were not abnormal when compared with controls. Group differences in thalamo-cortical and fronto-parietal functional connectivity were not observed. The strength of right caudate functional connectivity correlated with information processing, memory and executive abnormalities, which are hallmarks of the chronic effects of TBI (Colantonio *et al.*, 2004). Reduced functional connectivity was particularly observed between caudate subdivision and the cingulate cortex. In keeping with this reduction in functional connectivity, the structural integrity of the white matter tract connections from the caudate to ACC was reduced. This also correlated with processing speed and executive impairments, although this relationship was also seen with other damaged white matter tracts. The results suggest that disruption of fronto-caudate interactions may underpin common cognitive impairments seen in TBI and provide an anatomical target for treatment interventions.

Our results build on previous work showing that TBI often produces disruption of large-scale brain network activity that is relevant to cognition (Sharp *et al.*, 2014). Diffuse axonal injury often damages white matter tract connections between network nodes. This disrupts network function, leading to cognitive impairment (Sharp *et al.*, 2011). Previously, this has mainly been studied by investigating cortico-cortical interactions, but cortical-subcortical connectivity is likely to be key to many of the cognitive impairments seen after TBI. Abnormalities of thalamo-cortical pathways have been described, particularly in severely affected patients (Schiff *et al.*, 2007; Schiff, 2010; Giacino *et al.*, 2014). However, in other diseases abnormal corticostriatal interactions underpin cognitive and motor impairments. The striatum may be particularly vulnerable to the downstream effects of axonal injury as it receives the majority of cortical projections to the basal ganglia (Haber, 2003) and as it combines both cortical and subcortical information its interactions may be particularly important for the efficient functioning of large-scale brain networks (Bell and Shine, 2016). Although TBI typically has a complex effect on structural and functional connectivity (Sharp *et al.*, 2014), in this population we did not observe a reduction in functional connectivity in a number of control thalamo-cortical or fronto-parietal analyses. Furthermore, interindividual variability in the interactions between the thalamus and the ACC did not correlate with cognitive performance. Taken together, these results suggest that change in corticostriatal connectivity is particularly important in producing executive dysfunction after TBI, although it is important to consider this change as one part of a more distributed cortical and subcortical network that is involved in supporting complex cognitive functions.

The most striking relationship between caudate functional connectivity and behaviour was in the domain of executive function, although measures of information processing speed and memory function also showed relationships. Executive functions encompass various cognitive control processes, including information processing, conflict monitoring and action control (Heyder *et al.*, 2004). Executive dysfunction underpins many characteristic problems seen after TBI, such as behavioural disinhibition and altered personality (Damasio *et al.*, 1994). However, the neural basis for these problems remains unclear. Corticostriatal structural and functional pathways link prefrontal and anterior cingulate cortices through the striatum into the basal ganglia (Kunishio and Haber, 1994; Haber and Knutson, 2010) and have long been implicated in executive control processes (Heyder *et al.*, 2004; Shenhav *et al.*, 2013). Resting-state corticostriatal functional connectivity is correlated with executive performance in both healthy subjects and in diseases such as schizophrenia, Huntington’s disease and ADHD (Mennes *et al.*, 2011; Unschuld *et al.*, 2012; Su *et al.*, 2013; Gordon *et al.*, 2015). Future work could investigate in a more precise way how abnormal caudate interactions produced by TBI relate to specific patterns of executive impairment. Event-related functional MRI studies are likely to be informative for identifying dynamic changes in functional connectivity and their relationship to moment-to-moment changes in executive control.

We also demonstrated that caudate connectivity correlated with levels of fatigue; however overall, there were fewer correlations with functional outcome measures compared to neuropsychological assessments. This may be because of self-report measures being inherently more susceptible to confounds such as a lack of insight and response bias.

Although similarities are present between certain corticostriatal networks, a caudate-specific pattern of functional connectivity was identified and delineates an ‘executive control’ network, including areas such as the thalamus, anterior cingulate, and lateral prefrontal cortex. These networks associated with bilateral caudate were disrupted in TBI, whereas putamen connectivity was unaffected. This is similar to functional connectivity changes seen in Huntington’s disease, a disorder initially characterized by degeneration and altered connectivity of the caudate and subsequent executive dysfunction (Unschuld *et al.*, 2012). Importantly, despite the inherent heterogeneity of injury mechanism and pathology, we demonstrate a similar disruption in caudate connectivity across our TBI cohort. This common effect may result from the striatum’s central role as part of a subcortical hub of information processing and integration (Bell and Shine, 2016), rendering it particularly vulnerable to disruption irrespective of aetiology. Although the influence of the caudate for cognitive function needs to be considered in the context of a more distributed brain network, the results suggest that diverse patterns of brain injury converge to produce a common and cognitively important disruption to caudate interactions.

Previous research has shown that the caudate is specifically associated with executive function (Grahn *et al.*, 2008), with a right-lateralized system particularly involved (Casey *et al.*, 1997; Hart *et al.*, 2013). For example, in ADHD where impairments in executive function and attention are also prevalent, a right cortico-caudate network has been implicated, including areas such as the inferior frontal cortices, insula, dorsal anterior cingulate and caudate nucleus (Mennes *et al.*, 2011; Hart *et al.*, 2013). In addition, caudate activity during an executive function task has been shown to be associated with prefrontal measures of structural connectivity (Casey *et al.*, 2007).

The dorsal ACC plays a central role in cognitive control and the regions dense connectivity with the caudate suggest its interactions with the striatum is important for this function (Kemp and Powell, 1970; Yeterian and Van Hoesen, 1978). Functional imaging has shown the involvement of the ACC during the exertion of cognitive control. For example, the region robustly activates when conflict exists between potential actions, as exemplified by the Stroop and other similar tasks (Kerns *et al.*, 2004; Ham *et al.*, 2013). The region has been implicated in a range of processes, including reward processing, conflict monitoring, and action selection (Shenhav *et al.*, 2013). The ACC may play a particular role in the allocation of control based on the estimated value of that control (Heyder *et al.*, 2004; Shenhav *et al.*, 2013), with interactions between the striatum and the ACC perhaps important for adjusting behaviour through biasing response selection as a result of this evaluation (O'Reilly and Frank, 2006; Shenhav *et al.*, 2013; Wiecki and Frank, 2013). These observations are clinically relevant. In addition to failures of cognitive control in Huntington’s disease, children with ADHD fail to activate the ACC during performance on the Stroop task, suggesting that their cognitive impairments may result from a loss of ACC input during the inhibition of routine responses (Bush *et al.*, 1999). Our results suggest that disruption to connectivity between the dorsal ACC and the caudate leads to the inability to execute cognitive control appropriately, resulting in disordered behaviour and disability.

There are a number of potential causes for altered functional connectivity following TBI. It is possible that changes in functional connectivity reflect the pattern of structural brain injury, which might damage either grey or white matter. One mechanism is that structural injury might directly injure the fronto-striatal white matter tracts that connect cortical and subcortical structures. In keeping with this mechanism is the observation that widespread changes in white matter structure were found in the TBI patients. Altered fractional anisotropy and mean diffusivity was seen in the caudate to ACC connections as well as striatal connections to the motor cortex and in the corpus callosum. White matter tract structure was also correlated with information processing and executive function measures in a number of tracts suggesting a more general relationship with executive function impairment than that of functional connectivity. This suggests that diffuse axonal injury may lead to more specific functional changes in connections implicated in executive function, although this was not a specific relationship as altered white matter integrity was widespread and correlations with executive function were observed in other areas.

Focal brain lesions might also contribute to the functional connectivity abnormalities observed. One possibility is that the presence of focal lesions might confound the functional connectivity analysis through a direct effect of damaged brain tissue in the regions where functional connectivity was calculated. We examined this issue in part by checking whether the location and extent of these lesions were contributing to group differences in functional connectivity. There was only minimal spatial overlap between focal lesions and areas of altered caudate functional connectivity. Furthermore, when subjects with lesions in areas of functional connectivity change were completely removed, we still observed a strong relationship between functional connectivity and executive dysfunction in TBI patients, providing strong evidence that the results are not directly produced by the effects of focal lesions. A more interesting possibility is that a remote effect of focal lesions might be seen i.e. a type of diaschesis. Our analysis does not resolve this issue, as patients with focal lesions remote from the areas included in the functional connectivity analysis were included in all analyses.

The starting point for our analysis was data from the HCP study of healthy subjects, acquired using the most advanced MRI acquisition protocols. This has high spatial and temporal resolution providing an optimal starting point for analysis. This allowed us to identify distinct functional subdivisions of the striatum in a data-driven way, which has not previously been possible using lower resolution data. The striatal subdivisions identified in this way obeyed anatomical boundaries, such as the separation by laterality and anterior/posterior dimensions in the caudate. The subdivisions were spatially separated and robustly reproduced across different dimensionalities, suggesting that the functional decomposition used to investigate the effects of TBI is representative of the underlying data.

There are a number of potential limitations for our study. One factor that it is important to consider is the effect of motion on functional connectivity measures. A number of steps were taken to ensure that movement-induced biases did not affect the results. Based on strict exclusion criteria, several subjects (patients and controls) were removed entirely from the imaging analyses. After this step, minimal motion was present in both groups with no evidence of group differences. Furthermore, robust motion artefact removal was performed, leaving derived functional connectivity measures that were uncorrelated with movement. As is common in many studies on TBI, only a small number of females were recruited. This reflects the gender split that is seen in the TBI population, as males are at higher risk of TBI than females. Although gender differences could not be investigated directly, control and patient groups were gender-matched, ensuring any gender effects did not influence the results. Future work should specifically assess how gender affects outcome following TBI. Another potential confound is the influence of age on white matter integrity. However, this is unlikely to have had a significant impact on the results as age ranges across groups were well matched and age was included as a covariate in the analyses. A further potential limitation of this study is the moderate sample size of the control group. This may have an impact on our ability to identify brain–behaviour relationships in the control population, in part because of lower variability in the experimental measures observed across the control population. It is likely to be informative to explore the relationship between caudate connectivity and executive function in a larger cohort of healthy individuals.

In summary, we used an advanced data-driven approach to study the effects of TBI on cortico-striatal connectivity. Abnormalities of cortico-striatal connectivity were observed, with a breakdown in interactions between the right caudate and ACC strongly associated with executive dysfunction after TBI.

## Supplementary Material

Supplementary Table S1

Supplementary Table S2

Supplementary Data

Supplementary Figures 1 and 2

## Abbreviations

ACC: anterior cingulate cortex
HCP: Human Connectome Project
mICA: masked independent components analysis
TBI: traumatic brain injury
