# Supplementary material for: Altered caudate connectivity is associated with executive dysfunction after traumatic brain injury
Source: Brain. 2017 Nov 23;141(1):148–64. doi: 10.1093/brain/awx309 (PMC5837394; doi:10.1093/brain/awx309)
Supplement: Supplementary Table S1 [file brain-2017-00481-file012_awx309.pdf]

Study ID	Age	Gender	Time since Injury (months)	Length of PTA (Days)	Cause of Injury	Lowest GCS	Length of LOC	Initial CT findings	Initial MRI Findings	GOSE	Past Medical History	Medications at Visit
002	65	M	141	60	RTA	Unknown	Unknown	SDH: Right; Contusion: Right Temporal Lobe, Right Frontal Lobe, Left Frontal Lobe	Atrophy: Right Temporal Lobe; Contusion: Right Temporal Lobe, Left Inferior Frontal Lobe, Left Temporal Pole, Right Occipital Lobe; Microhaemorrhage: Left Superior Frontal Gyrus	6	None	None
005	38	M	49	3	Violence	Unknown	1-29 minutes	Fracture: Right parietal; Contusion: Right temporal; SAH	Superficial Siderosis: Right Frontal, Right Temporal; Microbleed: Right Frontal	8	None	None
006	51	F	37	2	Incident/ Fall	15	1-29 minutes	Contusion: Right Temporal Lobe; SAH; Fracture: Right Occipital Bone	Normal	6	Diverticular Disease	Omeprazole
007	31	M	46	60	RTA	3	> 7 Days	Unknown (in Dominican Republic)	Contusion: Left Frontal, Right Frontal, Left Occipital, Right Occipital, Left Temporal, Right Temporal, Corpus Callosum; Atrophy: Global	6	None	Propranolol 80mg
008	54	M	18	5	RTA	Unknown	30-59 minutes	Unknown	Contusion: Right Frontal, Left Frontal; Microhaemorrhages: Extensive, throughout	5	Hypothyroid	Citalopram 30mg, Thyroxine 150mcg
009	20	M	24	30	Incident/ Fall	Unknown	Unknown	Unknown	Microhaemorrhage: Left Temporal, Right Temporal, Left dorsal lentiform nucleus; Atrophy: Left Cerebral Peduncle; Additional; Signal change in left corticospinal tract	6	None	None

010	37	M	35	10	Violence	7	Unknown	Contusion: Left Temporal Lobe, Left Frontal Lobe; Fracture: Right Parietal; SAH: Left Frontal Lobe, Left Parietal Lobe, Left Temporal Lobe	Contusion: Left Temporal Lobe, Left Frontal Lobe; Microhaemorrhage: Right Temporal	6	None	None
011	44	M	42	90	RTA	Unknown	Unknown	Unknown	Microhaemorrhage: Parafalcine, Left Frontal, Right Frontal, Left Occipital, Right Occipital, Right Temporal, Subcortical	5	None	Amtripyline 40mg, Vitamin D, Lanzoprazole 15mg, Testosterone injections
012*	51	M	266	21	RTA	Unknown	1-29 minutes	Fracture: Occipital bone; Contusions: Left Frontal Lobe, Right Frontal Lobe	Contusion: Left Frontal Pole, Right Frontal Pole, Left Temporal Lobe	6	None	None
013	64	M	14	4 hours	RTA	14	0 - 1 Minute	SDH: Right; Fracture: Occipital Bone; SAH: Left Frontal; Contusion: Left Frontal, Right Frontal	Superficial Siderosis: Left Frontal Pole, Right Frontal Pole, Right Temporal Lobe; Contusion: Right Frontal Lobe, Left Frontal Lobe	6	Hypothyroid, Glaucoma	Omeprazole 40mg, Levothyroxine 75 mcg.
014	57	M	23	28	RTA	Unknown	0-1 minute	Unknown (in Lebanon)	Atrophy: Right Medial Temporal Lobe; Contusion: Right Cingulate Gyrus. Superficial siderosis: Parietal lobes	6	None	Aspirin
015	33	M	144	42	Incident/ Fall	3	> 7 Days	Unknown (in Zimbabwe)	Contusion: Right Occipital Lobe; Atrophy: Global; Microhaemorrhages: Left Frontal Parafalcine, Left medial Thalamus, Right Substantia Nigra, Right Temporal Lobe, Left Temporal Lobe	6	None	None
016	46	M	37	15	RTA	Unknown	1-29 minutes	SAH: Left Sylvian Fissure	Microbleeds: Parafalcine, Left Frontal, Right Frontal	7	Gastro-oesophageal relux disease	None

017*	20	M	23	60	RTA	13	1-29 minutes	SAH; Fracture: Left Occipital Bone; Contusion: Left Frontal, Right Frontal, Left Temporal, Right Temporal, Left Parietal, Right Parietal; SDH: Right Parieto-Temporal	Contusion: Right Temporal Pole, Right Frontal Pole; Microhaemorrhage: Left Parietal Lobe, Left Frontal Lobe, Right Frontal Lobe, Left Temporal Lobe, Left Occipital Lobe	5	None	None
021	52	M	43	5	RTA	Unknown	Unknown	Normal	Microhaemorrhage: Parafalcine, Left Frontal, Right Frontal	6	None	None
022	45	M	32	14	Other Non Intentional	15	0 - 1 Minute	Not done	Normal	6	None	None
025	31	M	73	90	Violence	Unknown	Unknown	Unknown: Multiple skull #s, intracranial haemorrhage, doffuse axonal injury	Contusion: Left Temporal Pole, Right Temporal Pole; Atrophy: Cerebellum; Superficial Siderosis: Cerebellum; Microhaemorrhage: Right Temporal Lobe, Left Frontal Lobe	5	None	None
026	49	M	249	120	Other Non Intentional	3	None	Unknown (in Bahamas)	Atrophy: Global, cerebellar, brain stem; Contusion: Right Frontal, Left Frontal, Right Temporal; Microhaemorrhage: Right Frontal, Left Frontal, Right Temporal	5	Rosacea	Sildenafil PRN, Loperamide PRN
028	24	M	8	14	RTA	Unknown	Unknown	Unknown	Microhaemorrhage: Parafaalcine, Right Frontal, Left Frontal, Left Parietal, Left lentiform nucleus, Left Temporal, Right Temporal	6	None	None

033*	52	M	17	21	Incident/ Fall	Unknown	1-29 minutes	SDH: Large Left with midline shift; SAH: Bilateral; Contusion: Left Frontal, Right Occipital; Fracture: Occipital bone, Sphenoid bone	Contusion: Left Temporal Lobe, Left Frontal Lobe, Right Frontal Lobe, Left Cerebellar Hemishere; Microhaemorrhage: Left Temporal, Left Frontal, Right Temporal, Right Frontal; Superficial Siderosis: Left Frontal, Left Temporal	6	None	Levetirecetam 1250mg BD, Perindopril 2mg OD
037*	54	M	11	15	Violence	12	1-24 Hours	SDH: Right; SAH: Left Frontal, Right Frontal; Contusion: Left Frontal, Left Temporal, Right Temporal, Right Occipital	Contusion: Right Temporal, Right Parietal, Right Frontal, Left Frontal, Left Temporal; Superficial Siderosis: Left Frontal	5	None	None
038	26	M	17	56	Violence	Unknown (in Romania)	None	Contusion: Left Frontal, Left Temporal, Left Parietal; SAH: Left Parietal; SDH: Left	Contusion: Left Frontal, Left Parietal; Superficial Siderosis: Left Parietal, Left Frontal	5	None	Amitriptylline 10mg OD, Sodium Valproate 100mg OD
039	21	F	14	35	RTA	Unknown	Unknown	Normal	Microhaemorrhage: Left Frontal, Corpus Callosum, Right Frontal	6	None	Fluoxetine 20mg, Propranolol 10mg PRN
040	39	F	6	2	Incident/ Fall	Unknown	0 - 1 Minute	SDH	Normal	6	None	None
041	54	M	24	1	Incident/ Fall	10	Unknown	Contusion: Left Temporal Lobe; SAH: Right Parietal, Left Temporal; Fracture: Left Squamous Temporal Bone	Contusion: Left Temporal Lobe; Microhaemorrhage: Right Temporal	8	Inflammatory Bowel Disease	Vitamin D
042*	47	M	51	3	RTA	Unknown	Unknown	SAH: Bilateral; SDH: Right Frontal, Right Parietal, Left Frontal, Left Parietal; Fracture: Right Temporal Bone	Contusion: Right Parietal Lobe; Microhaemorrhage: Subcortical; Superficial Siderosis: Right Parietal Lobe, Left Frontal Lobe	6	None	Atorvastatin 10mg, Growth Hormone

044	22	M	21	540	RTA	3	> 7 Days	SAH: Right Frontal, Left Frontal; Contusion: Splenium, Left Temporal	Contusion: Left Frontal, Right Frontal, Splenium, Left Thalamus, Left Temporal; Atrophy: Global; Microhaemorrhage: Subcortical, Parafalcine, Left Temporal, Right Temporal, Splenium, Right Frontal, Left Cerebellar	4	None	None
045*	34	M	6	2 hours	Incident/fal l	Unknown	0-1 minute	Unknown	Contusion: Small Bifrontal and Left Temporal Pole	6	None	None
046	48	M	366	60	RTA	3	> 7 Days	Unknown	Contusion: Parafalcine, Left Frontal, Right Frontal; Microhaemorrhage: Left Temporal	4	Arthritis	Amitrptylline 20mg TDS, Fexofenadine 180mg OD, Esomeprazole 40mg OD, Tramadol 50 mg TDS, Calcichew 1BD, Solifenacin 5mg TDS, Alvarine citrate 400mg BD, Botox injection 10mg OD, Mebeverine 500mg TDS, Loperamide 600mg TDS, Naproxen, Gabapentin 600mg TDS
047	38	M	210	720	Violence	3	> 7 Days	Unknown	Contusion: Left Occipital; Atrophy: Cerebellar, Pons, Left hemisphere	5	None	None
048	49	F	158	7	Violence	Unknown	1-29 minutes	Unknown	Normal	5	Hypothyroid	Levothyroxine 150 mcg, Omeprazole, Gamolenic acid, Pyridoxine
052	36	F	219	120	RTA	Unknown	Unknown	Unknown	Contusion: Right Temporal Lobe; Microhaemorrhage: Right Frontal Lobe, Posterior Limb Right Internal Capsule, Right Occipital Lobe; Atrophy: Cerebellum, Pons	4	None	None

053*	43	M	6	42	RTA	3	Unknown	SDH: Right; Contusion: Right Frontal Lobe	Superficial Siderosis: Right Frontal Lobe (vertex); Microhaemorrhage: Right Frontal lobe, Corpus Callosum (Genu and Splenium), Mid Brain.	5	None	None
054	31	M	22	21	Violence	Unknown	1-29 minutes	Contusion: Right Frontal, Right Temporal Lobe	Contusion: Right Frontal, Right Temporal Lobe	8	None	Lamotrigine 50mg BD, Growth Hormone 2 mg OD.
055	37	M	205	120	Incident/ Fall	3	> 7 Days	Unknown	Contusion: Right Frontal Lobe, Left Frontal Lobe, Left Temporal Lobe; Atrophy: Corpus Callosum, Left Temporal Lobe, Right Cerebral Peduncle; Previous Right Frontal Craniectomy	5	None	Tegetrol CR 400mg BD
056	33	M	147	28	RTA	Unknown	Unknown	Fracture: Left Squamous Temporal Bone; Contusion: Right Frontal Lobe, Left Frontal Lobe, Left Temporal Lobe; SAH: Left Frontal, Left Temporal Lobes	Contusion: Right Frontal Lobe; Microhaemorrhage: Parafalcine, Right Frontal, Left Frontal; High Signal: Periventricular and Pontine, inkeeping with demyelination	6	None	Baclofen 20mg OD, Citalopram 10mg OD
057*	34	M	9	7	Incident/ Fall	10	1-24 Hours	Normal	Contusion: Left Caudate Head; Microhaemorrhage: Left Temporal Pole, Left Occipital Lobe, Parafalcine (Left and Right Frontal Lobes)	6	None	None

060	32	M	73	28	RTA	3	> 7 Days	EDH: Right Temporal; Fracture: Right Temporal Bone, Right Greater Wing of Sphenoid, Left Frontal Bone, Left Greater Wing of Sphenoid, Left Maxillary Wall, Left Zygomatic Arch; SAH: Right Vertex	EDH: Left Frontal, Right Temporal, Left Occipital; SAH: Right Vertex; Contusion: Left Frontal Lobe; High Signal: Splenium; Microhaemorrhage: Left Frontal Lobe, Right Frontal Lobe	6	Asthma	Symbicort
061	52	M	23	4	Violence	Unknown	Unknown	Unknown	Contusion Right Frontal Lobe, Left Frontal Lobe, Left Temporal Lobe, Right Temporal Lobe	6	Gastro-oesophageal relux disease	Lanzoprazole 15 mg
063	38	M	9	14	Violence	3	1-24 Hours	Contusion: Left Frontal Lobe, Right Frontal Lobe; SDH: Parafalcine; SAH: Parafalcine; Fracture: Right Occipital Bone	Contusion: Right Frontal Lobe, Right Temporal Lobe; SDH: Parafalcine; SAH: Parafalcine	6	None	None
064	31	M	112	120	RTA	6	> 7 Days	Contusion: Left Frontal Lobe, Right Frontal Lobe, Corpus Callosum, Right Thalamus	Contusion: Parafalcine (Right and Left Frontal Lobes), Right Thalamus; Microhaemorrhage: Left Frontal Lobe, Right Frontal Lobe, Right Temporal Pole	5	None	None
065	39	M	33	8	RTA	Unknown	1-24 Hours	Contusion: Left Frontal Lobe	Contusion: Left Frontal Lobe, Right Frontal Lobe	5	None	None

Supplementary Table 1. Clinical demographics of the traumatic brain injury patients. PTA = Post-traumatic amnesia; GCS = Glasgow Coma Scale; LOC = Loss of Consciousness; CT = Computed Tomography; SDH = subdural haematoma; SAH = subarachnoid haemorrhage; RTA = Road Traffic Accident; OD = omni die (once a day); BD = bis die (twice a day); TDS = ter die sumendus (three times a day); QDS = quarter die sumendus (four times a day); PRN = pro re nata (taken if needed). *Study ID indicates those subjects whose lesions overlapped with areas of functional connectivity change.
