# Supplementary material for: Altered caudate connectivity is associated with executive dysfunction after traumatic brain injury
Source: Brain. 2017 Nov 23;141(1):148–64. doi: 10.1093/brain/awx309 (PMC5837394; doi:10.1093/brain/awx309)
Supplement: Supplementary Table S2 [file brain-2017-00481-file013_awx309.pdf]

		Thalamic 'Prefrontal' Region to ACC FC Rho (p-value)		Thalamic 'Primary Motor' Region to ACC FC Rho (p-value)	
**Cognitive Domain**	**Neuropsychological Test**	**Right**	**Left**	**Right**	**Left**
**Processing Speed**	Trail Making Test B (s)	0.294 (0.082)	0.233 (0.172)	0.229 (0.179)	0.281 (0.100)
	Stroop Colour Naming & Word Reading Composite Score (s)	-0.017 (0.921)	-0.090 (0.599)	0.074 (0.667)	0.054 (0.753)
**Executive Function**	Stroop Inhibition (s)	-0.002 (0.991)	-0.094 (0.587)	0.115 (0.506)	0.025 (0.883)
	Stroop Inhibition-Switching (s)	0.150 (0.382)	0.046 (0.789)	0.178 (0.298)	0.127 (0.460)
	Stroop Inhibition-Switching vs Baseline Contrast (s)	0.181 (0.291)	0.078 (0.651)	0.177 (0.303)	0.131 (0.450)
**Memory**	PT Delayed Recall	0.113 (0.511)	0.197 (0.249)	0.038 (0.826)	0.043 (0.803)

Supplementary Table 2. Relationship between neuropsychological measures and thalamic to anterior cingulate cortex (ACC) functional connectivity (FC) in TBI patients, using Spearman's rank-order approach (Rho). Peoples test (PT). Seconds (s).
