# Supplementary material for: Altered caudate connectivity is associated with executive dysfunction after traumatic brain injury
Source: Brain. 2017 Nov 23;141(1):148–64. doi: 10.1093/brain/awx309 (PMC5837394; doi:10.1093/brain/awx309)
Supplement: Supplementary Data [file brain-2017-00481-file010_awx309.pdf]

Supplementary Material

Supplementary Methods

Structural and functional magnetic resonance imaging data acquisition

Each participant's resting-state fMRI (rfMRI) data from the Human Connectome Project (HCP) dataset included one 15-minute run (rest 1) with left-to-right phase encoding (2mm isotropic, TR = 0.7s). Diffusion-weighted volumes were acquired using a multi-shell 90-direction protocol (b-value = 1000/2000/3000mm^2.s^{-1}, 1.25mm isotropic, TR = 5.5s). Six non-diffusion weighted B0 images were acquired twice, to enable opposing phase encoding directions. For details on acquisition procedures please see (Glasser, Sotiropoulos, *et al.* , 2013; Smith, Beckmann, *et al.* , 2013; Sotiropoulos, Jbabdi, *et al.* , 2013; Van Essen, Smith, *et al.* , 2013).

MRI data from a separate cohort of TBI patients and healthy controls, were acquired using a 3T Siemens Magnetom Verio Syngo using a 32-channel head coil. Standard clinical MR imaging was collected, including a T2-weighted inversion recovery turbo spin echo (IR-TSE) (30 4-mm-thick transverse slices, TR = 9000 ms, TE = 95 ms, FA = 130°, matrix size = 256 x 256, field of view = 22 x 22 cm) and T2* susceptibility-weighted (SWI) scans (128 1.2-mm-thick transverse slices, TR = 28 ms, TE = 20 ms, FA = 15°, in-plane resolution = 0.6 x 0.5 mm, matrix size = 448 x 448, field of view = 23 x 23 cm) to assess microbleeds. Functional T2*-weighted rfMRI data were collected using gradient echo EPI. A total of 300 volumes of 35 slices (3 mm thickness, no interslice gap) were acquired in an interleaved manner during a 10-minute scan (TE = 30 ms, TR = 2000 ms, flip angle (FA) = 80°, in-plane resolution = 3 x 3mm, matrix size = 64 x 64, field of view = 19.2x19.2 cm). Participants were asked to keep their eyes closed during rfMRI.

Structural MRI included a T1-weighted high-resolution MPRAGE (160 1-mm-thick transverse slices, TR = 2300 ms, TE = 2.98 ms, FA = 9°, in-plane resolution = 1 x 1mm, matrix size = 256 x 256, field of view = 25.6 x 25.6 cm) and diffusion tensor

imaging. Diffusion-weighted volumes were acquired using a 64-direction protocol (64 slices, in-plane resolution = 2 x 2mm, slice thickness = 2mm, field of view = 25.6 x 25.6 cm, matrix size = 128 x 128, TR = 9500ms, TE = 103ms, b-value = 1000mm^2.s^{-1}). Four non-diffusion weighted images were also acquired (b-value = 0mm^2.s^{-1}).

Human Connectome Project (HCP) Analysis

Preprocessing

Pre-processing of the HCP rfMRI data included motion and distortion correction. rfMRI data was registered to Montreal Neurological Institute (MNI) standard space using a two-step process. The T1-weighted structural was non-linearly registered to standard space and concatenated with the rfMRI to T1 structural transform. Temporal filtering included minimal high-pass filtering (2000 seconds). Artefact removal was performed with the use of FMRIB's ICA-based X-noisifier (FIX) (Salimi-Khorshidi, Douaud, *et al.* , 2014; Griffanti, Douaud, *et al.* , 2016), which includes regressing out independent components (ICs) classified as noise and 24 motion parameter timeseries estimated through motion correction procedures. A subset of 94 healthy participants from the HCP was used for the tractography analysis. Preprocessing of the diffusion data included B0 intensity normalisation, estimation of EPI distortion with FSL TOPUP using B0 images acquired in opposing phase encoding directions, eddy current and head motion correction using the FSL EDDY tool, correction for gradient non-linearity and registration to T1-weighted structural space using BBR (Glasser, Sotiropoulos, *et al.*, 2013).

Clinical Study Data

Preprocessing

The first two volumes of the rfMRI were removed to account for the T1 equilibrium effect. rfMRI data preprocessing included a rigid-body realignment to correct for motion between volumes, temporal frequency filtering using a high-pass filter (100 seconds), followed by spatial smoothing using a 6 mm full-width at half-maximum (FWHM) kernel. Functional images were registered to standard MNI space using a

two-step procedure. The T1-weighted structural was linearly registered to standard space and concatenated with the rfMRI to T1 structural transform, performed using boundary-based registration (BBR; Greve & Fischl, 2009).

Wholebrain independent components analysis (ICA) was performed to identify spatially independent temporal components within each subjects' rfMRI data. As with the HCP, artefact removal was performed with FIX, previously trained on an independent dataset of 20 healthy controls. The accuracy of FIX classification was also assessed manually (Griffanti, Douaud, *et al.*, 2016). Prior to running FIX, movement during rfMRI was assessed using two measures, frame-wise displacement (FD) and intensity changes in the BOLD signal across the whole brain (DVARS). These were calculated through FSL (Motion Outliers) as per Power et al., (2012; 2014). Criteria for excessive motion included FD of > 0.5mm in more than 15% of volumes and/or an FD of > 3mm (size of one voxel) across any of the volumes (Carriere, Lopes, *et al.* , 2015; Manza, Zhang, *et al.* , 2016). Using these criteria, a subset of patients and controls were excluded from the imaging analysis (see Results: Control Analyses).

Diffusion data were corrected for head motion and eddy current distortions, using linear transformations to register these images to the b = 0 image. A brain mask was generated by brain extracting the b = 0 image (FSL Brain extraction tool; (Smith, 2002)). A tensor model was then fitted to the data using FMRIB's Diffusion Toolbox (FDT) in FSL, constrained by the brain mask. Applying this tensor model generated voxel-wise individual subject fractional anisotropy (FA), mean diffusivity (MD) maps. These maps were transformed into 1mm-resolution standard space using DTI-TK (Zhang, Yushkevich, *et al.* , 2006). An initial group based template was generated through bootstrapping of the tensor-based maps together with the predefined IXI aging standard template (Zhang, Yushkevich, *et al.* , 2010). Individual tensor-based images were then registered to the group template using diffeomorphic transformations.

Lesion Analysis

Focal lesions were manually defined as binary masks in the space of the native high-resolution T1, with the high-resolution T2 scan as a comparison. This was done using a customised software tool (IMSEG v1.8) for semi-automatic segmentation of medical images which is based on an algorithm for geodesic image segmentation as described in Criminisi, Sharp et al., (2008). Lesion masks were then registered to standard space using FLIRT (Jenkinson, Bannister, *et al.* , 2002). Registration parameters were defined by registering the participant's skull-stripped structural T1 image to the MNI152 2mm standard template. Lesion volumes were calculated using fslstats and overlap images were created using MRICroGL (Supplementary Fig.1C). Lesion locations were also reported by a neuroradiologist (Supplementary Table 1).

Supplementary Results

Abnormalities in FC were not primarily the result of lesioned cortex

As expected in TBI, focal lesions were primarily located in right orbitofrontal and left temporal cortical regions ((Gurdjian, 1975); Supplementary Fig.1C). 25 out of 36 TBI patients included in the imaging analysis showed evidence of focal lesions, although areas of greatest overlap were driven only by a maximum of ten patients (Supplementary Fig.1C). On visual inspection, the areas with high lesion overlap did not generally include areas of the brain with abnormal FC in the patients. To quantify the spatial separation between changes in caudate connectivity and lesion locations, pairwise correlations were performed between the FC results and the lesion overlap map. A maximum spatial correlation of 0.19 was found, suggesting that distinct brain regions were lesioned to those showing FC changes. Furthermore, the relationship between FC changes in the right anterior caudate and cognitive impairment, including information processing (Trail Making: rho=-0.447, p=0.017; Inhibition: rho=-0.537, p=0.003; Switching: rho=-0.588, p=0.001) and executive function (Inhibition-Switching vs Baseline Contrast: rho=-0.547, p=0.003), was maintained following the removal of eight patients whose lesions did overlap with areas of FC change (subjects identified with a * in Supplementary Table 1 were those excluded; Supplementary

Fig.1D). There was a wide range of lesion volumes (range of 66 mm^3–197818 mm^3, mean 36932.2 +/- 47275.9). Lesion volumes did not correlate with anterior caudate connectivity, but correlated with right posterior caudate connectivity ($p<0.05$).

References

Carriere N, Lopes R, Defebvre L, Delmaire C, Dujardin K. Impaired corticostriatal connectivity in impulse control disorders in Parkinson disease. Neurology 2015;84:2116-2123.

Criminisi A, Sharp T, Blake A. GeoS- Geodesic Image Segmentation. In: Forsyth D, Torr P, Zisserman A, eds. ECCV 2008 Part I: Springer-Verlag Berlin Heidelberg 2008: 99-112.

Glasser MF, Sotiropoulos SN, Wilson JA, Coalson TS, Fischl B, Andersson JL, *et al.* The minimal preprocessing pipelines for the Human Connectome Project. Neuroimage 2013;80:105-124.

Griffanti L, Douaud G, Bijsterbosch J, Evangelisti S, Alfaro-Almagro F, Glasser MF, *et al.* Hand classification of fMRI ICA noise components. Neuroimage 2016.

Gurdjian ES. Re-evaluation of the biomechanics of blunt impact injury of the head. Surg Gynaecol Obstet 1975;140:845-850.

Jenkinson M, Bannister P, Brady M, Smith S. Improved Optimization for the Robust and Accurate Linear Registration and Motion Correction of Brain Images. NeuroImage 2002;17:825-841.

Manza P, Zhang S, Li CS, Leung HC. Resting-state functional connectivity of the striatum in early-stage Parkinson's disease: Cognitive decline and motor symptomatology. Hum Brain Mapp 2016;37:648-662.

Power JD, Barnes KA, Snyder AZ, Schlaggar BL, Petersen SE. Spurious but systematic correlations in functional connectivity MRI networks arise from subject motion. Neuroimage 2012;59:2142-2154.

Power JD, Mitra A, Laumann TO, Snyder AZ, Schlaggar BL, Petersen SE. Methods to detect, characterize, and remove motion artifact in resting state fMRI. Neuroimage 2014;84:320-341.

Salimi-Khorshidi G, Douaud G, Beckmann CF, Glasser MF, Griffanti L, Smith SM. Automatic denoising of functional MRI data: combining independent component analysis and hierarchical fusion of classifiers. Neuroimage 2014;90:449-468.

Smith SM. Fast robust automated brain extraction. Hum Brain Mapp 2002;17:143-155.

Smith SM, Beckmann CF, Andersson J, Auerbach EJ, Bijsterbosch J, Douaud G, *et al.* Resting-state fMRI in the Human Connectome Project. Neuroimage 2013;80:144-168.

Sotiropoulos SN, Jbabdi S, Xu J, Andersson JL, Moeller S, Auerbach EJ, *et al.* Advances in diffusion MRI acquisition and processing in the Human Connectome Project. Neuroimage 2013;80:125-143.

Van Essen DC, Smith SM, Barch DM, Behrens TE, Yacoub E, Ugurbil K, *et al.* The WU-Minn Human Connectome Project: an overview. Neuroimage 2013;80:62-79.

Zhang H, Yushkevich PA, Alexander DC, Gee JC. Deformable registration of diffusion tensor MR images with explicit orientation optimization. Med Image Anal 2006;10:764-785.

Zhang H, Yushkevich PA, Rueckert D, Gee JC. A Computational White Matter Atlas for Aging with Surface-Based Representation of Fasciculi. International Workshop on Biomedical Image Registration, 6204, Lecture Notes in Computer Science 2010:83-90.
