# Supplementary material for: Altered caudate connectivity is associated with executive dysfunction after traumatic brain injury
Source: Brain. 2017 Nov 23;141(1):148–64. doi: 10.1093/brain/awx309 (PMC5837394; doi:10.1093/brain/awx309)
Supplement: Supplementary Figures 1 and 2 [file awx309_supplementary_figures_1_and_2.pdf]

Supplementary Figure 1. Evaluation of the striatal subdivisions and lesion overlap maps. (A) Correlation matrix representing the pairwise correlations between all of the twelve striatal subdivisions identified. The coloured bar represents the correlation coefficients. Blue colours indicate lower correlations. **(B)** Stability of the four caudate subdivisions across different independent component analysis (ICA) dimensionalities. The highest spatial correlation identified between these subdivisions and components within DIM2-DIM20 are plotted. Higher spatial correlations suggest that the striatal subdivisions derived from DIM12 can be robustly identified across other dimensionalities. This pattern was present across all striatal subdivisions derived from DIM12, not presented in the figure for ease of visualisation. **(C)** Overlap map of focal lesions defined on structural T1 and T2 images in traumatic brain injury (TBI) patients. The colour bar indicates the areas of lesion overlap. Red areas represent areas of highest overlap. L = left, R = right.

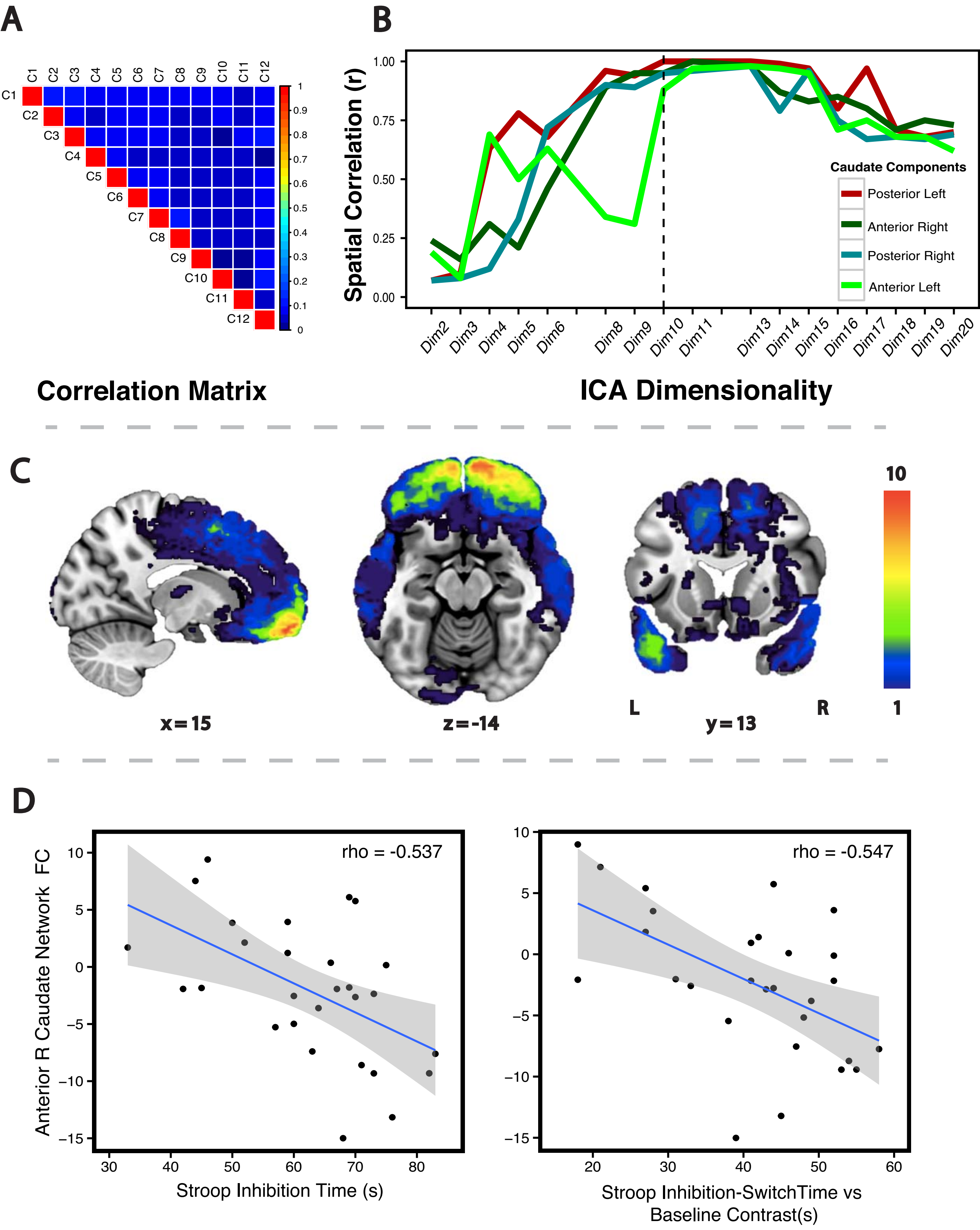

A
C1 C2 C3 C4 C5 C6 C7 C8 C9 C10 C11 C12
1 0.9 0.8 0.7 0.6 0.5 0.4 0.3 0.2 0.1 0
Correlation Matrix
B
Spatial Correlation (r)
1.00 0.75 0.50 0.25 0.00
Caudate Components
Posterior Left
Anterior Right
Posterior Right
Anterior Left
Dim2 Dim3 Dim4 Dim5 Dim6 Dim8 Dim9 Dim10 Dim11 Dim13 Dim14 Dim15 Dim16 Dim17 Dim18 Dim19 Dim20
ICA Dimensionality
C
x=15
z=-14
L
y=13
R
10
1
D
Anterior R Caudate Network FC
rho = -0.537
Stroop Inhibition Time (s)
rho = -0.547
Stroop Inhibition-SwitchTime vs Baseline Contrast(s)

Supplementary Figure 2. The relationship between thalamic to anterior cingulate cortex (ACC) functional connectivity (FC) and neuropsychological measures. (A) Four separate thalamic subdivisions were used to examine thalamo-cortical connectivity: blue/red subdivisions have high structural connectivity to the prefrontal cortex, yellow/green subdivisions have high connectivity to primary motor cortex. (B) Box plots showing thalamic-ACC FC in healthy controls (CON) and traumatic brain injury (TBI) (C) Scatter plots showing the relationship between thalamic-ACC functional connectivity and reaction times (seconds) for Stroop Inhibition-Switch time vs baseline, an executive measure corrected for baseline speed.

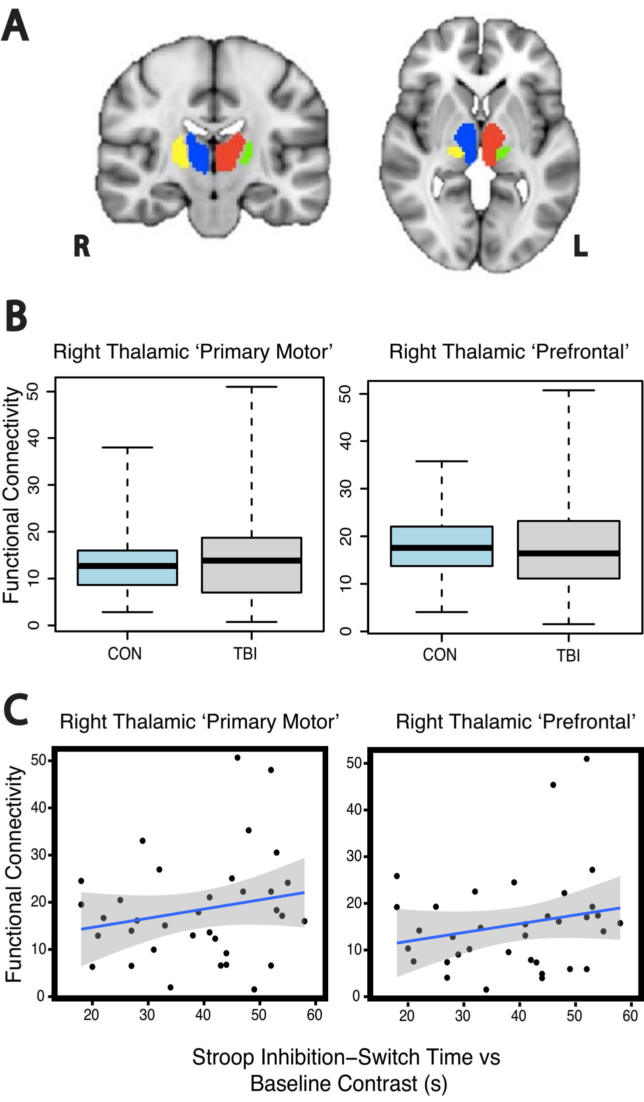
A
R
L
B
Right Thalamic 'Primary Motor'
Right Thalamic 'Prefrontal'
Functional Connectivity
0
10
20
30
40
50
CON
TBI
C
Right Thalamic 'Primary Motor'
Right Thalamic 'Prefrontal'
Functional Connectivity
20
30
40
50
60
Stroop Inhibition–Switch Time vs
Baseline Contrast (s)
